# A 70% Ethanol *Neorhodomela munita* Extract Attenuates RANKL-Induced Osteoclast Activation and H_2_O_2_-Induced Osteoblast Apoptosis In Vitro

**DOI:** 10.3390/molecules29081741

**Published:** 2024-04-11

**Authors:** Seongtae Jeong, Il-Kwon Kim, Hanbyeol Moon, Hojin Kim, Byeong-Wook Song, Jung-Won Choi, Sang Woo Kim, Seahyoung Lee, Dong-Sik Chae, Soyeon Lim

**Affiliations:** 1 The Interdisciplinary Graduate Program in Integrative Biotechnology, Yonsei University, Seoul 03722, Republic of Korea; 91seongtae@gmail.com; 2 Department of Convergence Science, College of Medicine, Catholic Kwandong University, International St. Mary’s Hospital, Incheon 22711, Republic of Korea; ilkwonkim@cku.ac.kr; 3 Department of Integrated Omics for Biomedical Sciences, Graduate School, Yonsei University, Seoul 03722, Republic of Korea; moonstar3636@naver.com; 4 Department for Medical Science, College of Medicine, Catholic Kwandong University, Gangneung-si 25601, Republic of Korea; blue_expanse@naver.com; 5 Department of Convergence Science, College of Medicine, Catholic Kwandong University, Gangneung-si 25601, Republic of Korea; songbw@ish.ac.kr (B.-W.S.); doctor7408@gmail.com (S.W.K.); sam1017@ish.ac.kr (S.L.); 6 Medical Science Research Institute, College of Medicine, Catholic Kwandong University, Incheon Metropolitan City 22711, Republic of Korea; gardinia@hanmail.net; 7 Department of Orthopedic Surgery, International St. Mary’s Hospital, Catholic Kwandong University, Gangneung-si 25601, Republic of Korea

**Keywords:** keyword ethanol extract of *Neorhodomela munita*, osteoclast, osteoblast, in vitro osteoporosis

## Abstract

The rapid aging of the population worldwide presents a significant social and economic challenge, particularly due to osteoporotic fractures, primarily resulting from an imbalance between osteoclast-mediated bone resorption and osteoblast-mediated bone formation. While conventional therapies offer benefits, they also present limitations and a range of adverse effects. This study explores the protective impact of *Neorhodomela munita* ethanol extract (EN) on osteoporosis by modulating critical pathways in osteoclastogenesis and apoptosis. Raw264.7 cells and Saos-2 cells were used for in vitro osteoclast and osteoblast models, respectively. By utilizing various in vitro methods to detect osteoclast differentiation/activation and osteoblast death, it was demonstrated that the EN’s potential to inhibit RANKL induced osteoclast formation and activation by targeting the MAPKs-NFATc1/c-Fos pathway and reducing H_2_O_2_-induced cell death through the downregulation of apoptotic signals. This study highlights the potential benefits of EN for osteoporosis and suggests that EN is a promising natural alternative to traditional treatments.

## 1. Introduction

Osteoporosis is the most prevalent metabolic disease in older adults, particularly those over the age of 50. It is characterized by reduced bone mass and the structural deterioration of bone tissue, resulting from an imbalance between osteoclast-mediated bone resorption and osteoblast-mediated bone formation. This imbalance leads to an increased prevalence of fractures, particularly in the lumbar spine, hip, and wrist, which are strongly correlated with increased mortality rates [1,2,3,4]. The rapid aging of the population in many countries poses a significant social and economic burden, especially in the case of osteoporotic fractures [5,6,7]. Aging, including menopause, is considered one of the most important risk factors, associated with chronic inflammation and immune system remodeling [8]. Under these conditions, an upregulation of pro-inflammatory cytokines such as Interleukin-1 (IL-1), IL-6, and tumor necrosis factor (TNF)-α has been observed, which are implicated in the osteoporosis process [9]. Cumulative results have shown that these cytokines can stimulate osteoclast differentiation and activation through the upregulation of T cells or the osteoblast-produced receptor activator of nuclear factor (NF)-κB ligand (RANKL) [10,11]. 

RANKL, a key stimulant of osteoclastogenesis, binds to RANK on hematopoietic precursor cells, leading to trimerization via the recruitment of its adaptor molecule, TNF receptor-associated factor 6 (TRAF6) [12]. TRAF6 then activates signaling molecules such as mitogen-activated protein kinases (MAPKs), nuclear factor-κB (NF-κB), and the tyrosine kinases c-Src and phosphatidylinositol 3-kinase (PI3K), inducing osteoclast differentiation and activation [13]. Another important transcription factor, activator protein-1 (AP-1), composed of Fos, Jun, and activating transcription factor (ATF), is also activated in the early stages of RANKL-induced signaling [14]. Activated NF-κB and AP-1 increase the expression of nuclear factor of activated T-cells cytoplasmic 1 (NFATc1), a key transcription factor that regulates gene expression in osteoclastogenesis, including tartrate-resistant acid phosphatase (TRAP), cathepsin K (CTSK), calcitonin receptor (CALCR), dendritic-cell-specific transmembrane protein (DC-STAMP), and osteoclast-associated receptor (OSCAR), which are directly involved in osteoclast differentiation and activation [15,16]. 

Extensive research on the mechanisms of osteoporosis has led to the development of various anti-resorptive drugs, such as bisphosphonates, RANKL antibodies (Denosumab), calcitonin, and selective estrogen receptor modulators (SERMs), as well as anabolic agents like parathyroid hormone. These are commonly used in clinical treatments for osteoporosis, either alone or in combination therapy [17,18]. However, these conventional therapies are associated with limited efficacy and a range of common and rare adverse effects, such as an increased risk of fractures [19,20], gastrointestinal and renal complications [21], and rhinitis [22], depending on the duration of the therapy or dosage. Consequently, natural compounds have been suggested as a promising alternative option to overcome the side effects of conventional therapy [23,24]. 

Marine algae are among the richest sources of diverse natural products and have been used as food or in traditional medicine [25,26]. Novel compounds derived from marine algae have demonstrated a variety of therapeutic potentials, including anti-tumor, anti-diabetic, anti-thrombotic, and other effects [27,28,29].

*Neorhodomela munita* (Perestenko) Masuda 1982, distributed along the coasts of Korea, Japan, and China, is a red algae belonging to the Rhodomelaceae family [30,31]. The therapeutic effects of *Neorhodomela*, including its antioxidative, anti-inflammatory, anti-bacterial, and anti-viral activities, have been reported for methanolic extracts of *Neorhodomela aculeata*, another species found in Korea [31,32]. However, the role of *Neorhodomela munita* extract in regulating metabolic bone disorders such as excessive osteoclastogenesis remains unexplored.

This study investigated the effects of an ethanol extract of *Neorhodomela munita* (EN) on RANKL-mediated osteoclast differentiation and activation, and the potential mechanisms underlying its anti-osteoclastogenic effects. Additionally, the protective effect of EN on H_2_O_2_-induced cell death in Saos-2 cells was examined.

## 2. Results

### 2.1. Subsection

#### 2.1.1. The Identification of Compounds of EN

LC-MS analysis was performed to identify the components of EN. The LC/MS chromatogram of EN is presented in Figure S1 and the identified components are listed in Table 1 and Figure S2, with the retention time and intensity. 

#### 2.1.2. EN Inhibits RANKL-Induced Osteoclast Differentiation and Activation of Raw264.7 Cells

**Figure 1:**
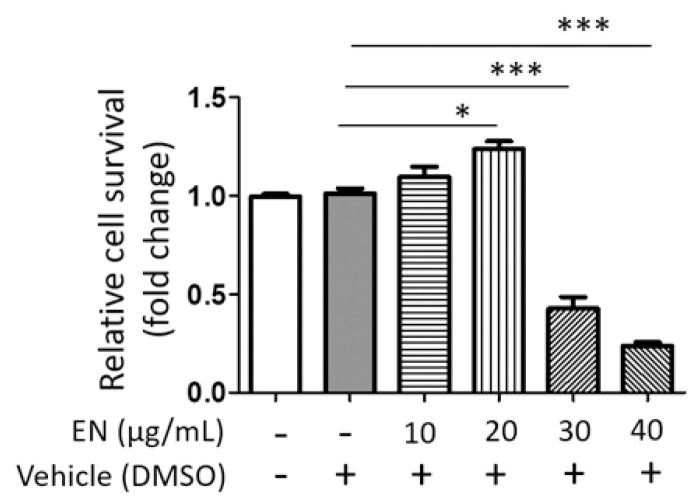
**Cell viability of *Neorhodomela munita* extract (EN) in Raw264.7 cells.** Relative cell survival rate was assessed in Raw264.7 cells treated with EN (10–40 μg/mL) on day 4 using the WST-1 assay. Results are expressed as mean ± SEM (*n* ≥ 3). For comparison between the vehicle and each EN treatment group, Student’s *t*-test was employed. *** *p* < 0.001; * *p* < 0.05 versus the vehicle treatment group; DMSO: dimethyl sulfoxide.

**Figure 2:**
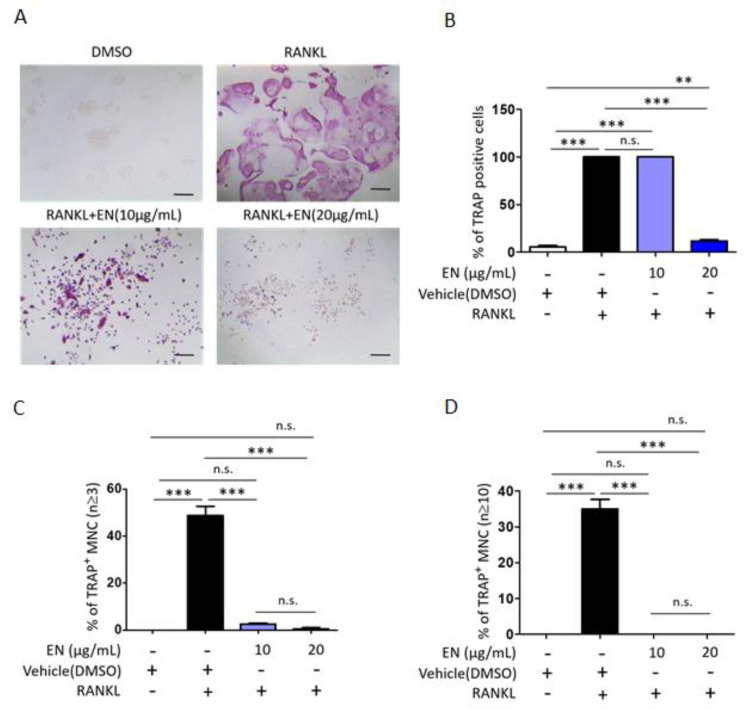
**EN attenuates RANKL-induced osteoclast differentiation.** Raw264.7 cells were pre-treated with EN (10 and 20 μg/mL) for 1 h, followed by RANKL (40 ng/mL) for 4 days. (**A**) EN’s inhibitory effect on osteoclast differentiation in Raw264.7 cells via TRAP staining shown in dark purple was observed at 40× magnification under a light microscope (Scale bar. 400 μm). (**B**–**D**) Counts of TRAP-positive cells, multinuclear cells (≥3 nuclei), and larger multinuclear cells (≥10 nuclei), presented as percentages. Data are shown as mean ± SEM (*n* ≥ 3). One-way analysis of variance (ANOVA) was utilized, followed by the Bonferroni post hoc test for pairwise comparisons. ** *p* < 0.01; *** *p* < 0.001; n.s. = not significant. DMSO: dimethyl sulfoxide.

**Figure 3:**
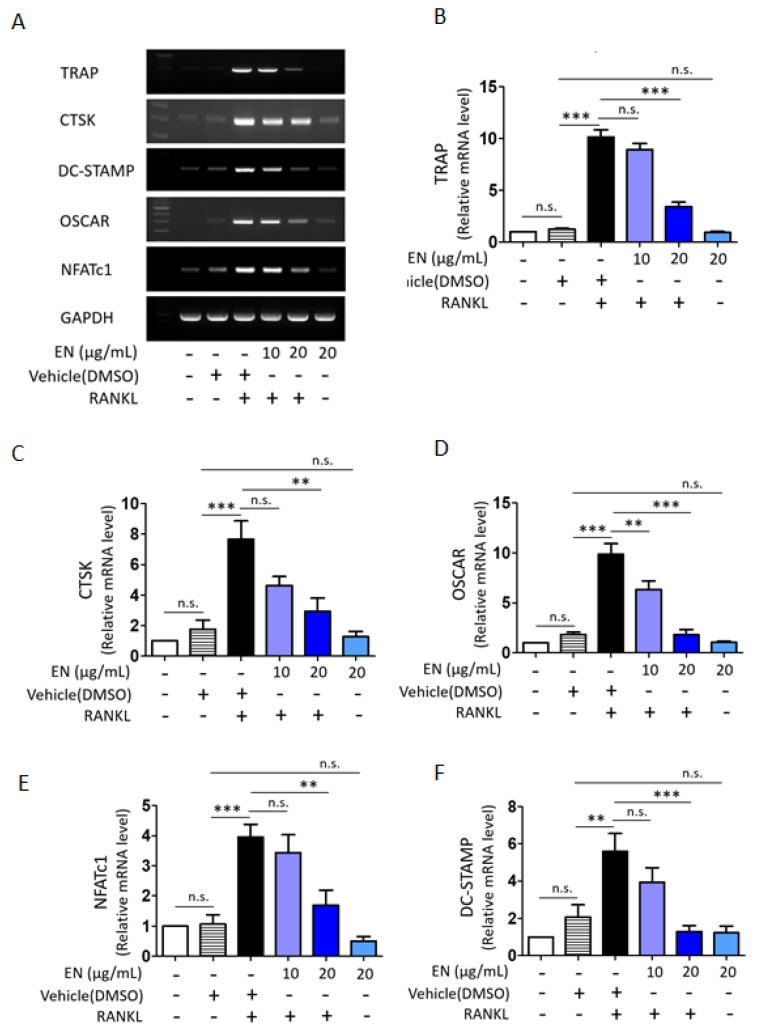
**Impact of EN on osteoclast-specific gene expression.** (**A**) The mRNA expression levels of osteoclast-specific genes were analyzed by RT-PCR in Raw264.7 cells treated with RANKL (40 ng/mL) with or without EN (10 and 20 μg/mL) for 2 days. (**B**–**F**) The RT-PCR results, normalized to GAPDH, for TRAP, CTSK, DC-STAMP, OSCAR and NFATc1 are presented in a bar graph. The quantitative data are expressed as mean ± SEM (*n* ≥ 3). One-way analysis of variance (ANOVA) was utilized, followed by the Bonferroni post hoc test for pairwise comparisons. ** *p <* 0.01; *** *p <* 0.001; n.s. = not significant. DMSO: dimethyl sulfoxide.

We tested the cytotoxic concentration of EN by using the WST-1 assay, and the results indicated that EN did not significantly reduce cell viability up to a concentration of 20 μg/mL (Figure 1). Subsequently, its protective effect was evaluated at concentrations of 10 and 20 µg/mL on RANKL-stimulated Raw264.7 cells. Tartrate-resistant acid phosphatase (TRAP), a key marker enzyme for osteoclasts, was stained to verify RANKL-induced osteoclast differentiation and the inhibitory effect of EN (Figure 2A). Quantitative analysis revealed that EN (20 µg/mL) significantly and concentration-dependently reduced the number of TRAP-positive cells (Figure 2B). RANKL induced giant multinucleated osteoclasts (48.75 ± 8.05% for *n* ≥ 3 and 35 ± 5.48% for *n* ≥ 10) and EN attenuated TRAP-positive multinucleated cells in a concentration-dependent manner (Figure 2C,D). Furthermore, EN decreased the RANKL-induced mRNA expression of osteoclast-specific genes, including TRAP, CTSK, DC-STAMP, and OSCAR, in a concentration-dependent manner (Figure 3A–D). The mRNA expression of NFATc1, a crucial transcription factor in osteoclastogenesis, was also downregulated by EN treatment under RANKL stimulation (Figure 3E).

#### 2.1.3. EN Impairs the Catabolic Functions of RANKL-Stimulated Osteoclasts

**Figure 4:**
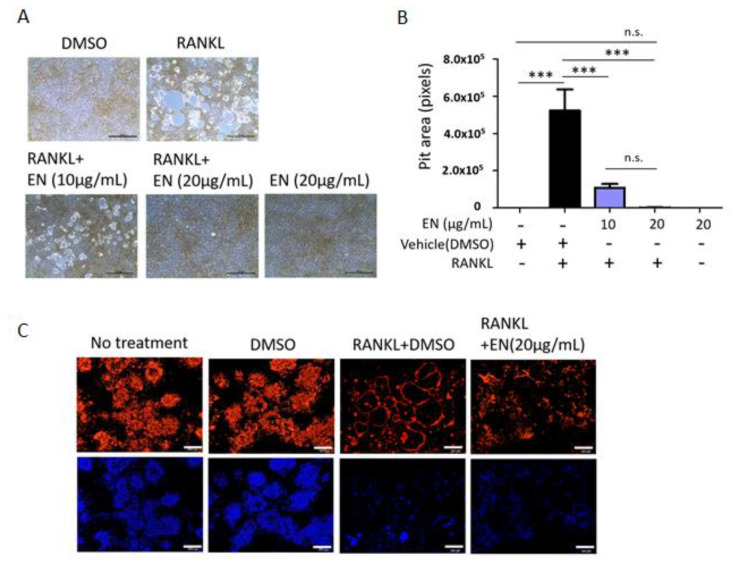
**EN’s role in osteoclast differentiation and function.** Raw264.7 cells were pre-treated with EN (10 and 20 μg/mL) for 1 h, followed by RANKL (40 ng/mL). (**A**) The area of resorption pits formed by mature osteoclast cells was evaluated using a bone resorption assay and visualized under a light microscope at 40× magnification (Scale bar. 100 μm). (**B**) The pit area measurements, quantified in pixels, are depicted in a bar graph. (**C**) The formation of actin rings in mature osteoclast cells was observed using a LSM 700 laser scanning confocal microscope, highlighting F-actin staining (upper line) and DAPI staining (bottom line) at 100× magnification (Scale bar = 200 μm). Data represent mean ± SEM (*n* ≥ 3). One-way analysis of variance (ANOVA) was utilized, followed by the Bonferroni post hoc test for pairwise comparisons. *** *p <* 0.001; n.s. = not significant. DMSO: dimethyl sulfoxide.

EN’s regulatory effect on essential osteoclast functions, such as bone resorption and F-actin ring formation, was assessed in RANKL-stimulated Raw264.7 cells. RANKL notably increased the pit area, depicted as white empty spaces, which was effectively reduced by EN at both 10 and 20 µg/mL concentrations, indicating a decrease in bone resorptive activity (Figure 4A,B). Consistently, EN also suppressed RANKL-induced F-actin ring formation, another marker of activated osteoclasts (Figure 4C) [33], demonstrating EN’s capability to disrupt the activation of RANKL-stimulated osteoclasts.

#### 2.1.4. EN Inhibits RANKL-Induced MAPK Signaling and Subsequent NFATc1 Activation

**Figure 5:**
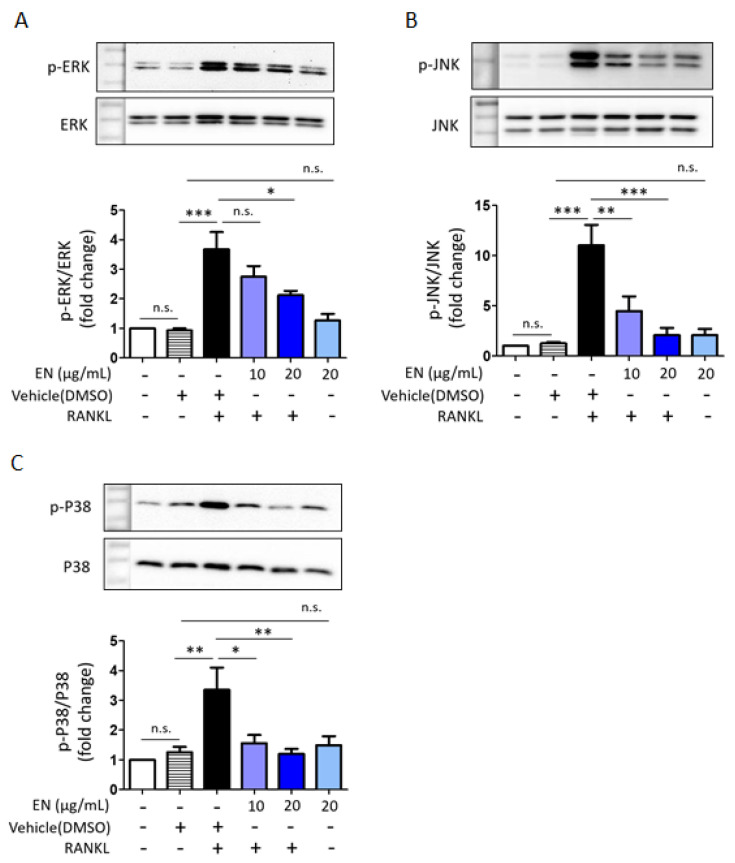
**EN modulates MAPKs signaling.** (**A**–**C**) Western blot analysis was used to determine the phosphorylation level of MAPKs in Raw264.7 cells pre-treated with EN (10 and 20 μg/mL) for 1 h, followed by RANKL (40 ng/mL) for 10 min. Protein expression levels were normalized to the expression levels of ERK, JNK, and p38, respectively. Data represent mean ± SEM (*n* ≥ 3). One-way analysis of variance (ANOVA) was utilized, followed by the Bonferroni post hoc test for pairwise comparisons. * *p* < 0.05; ** *p* < 0.01; *** *p* < 0.001; n.s. = not significant. DMSO: dimethyl sulfoxide.

**Figure 6:**
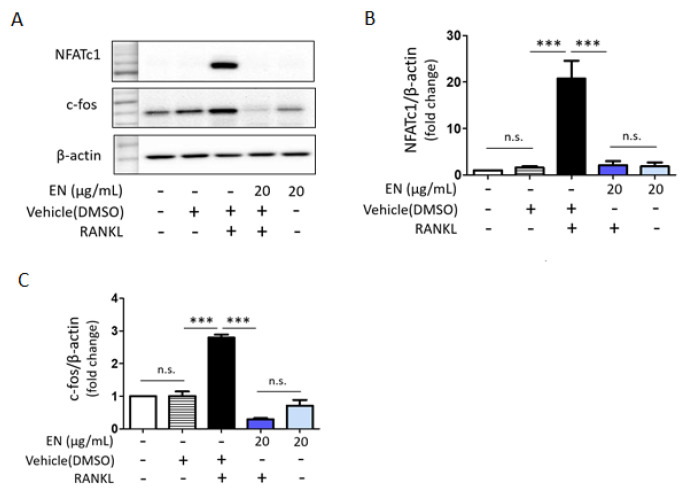
**EN’s effects on NFATc1 and c-fos post RANKL treatment.** (**A**) Western blot analysis was conducted to evaluate the expression levels of NFATc1 and c-fos in Raw264.7 cells 24 h post-treatment with RANKL (40 ng/mL). (**B**,**C**) The findings are displayed in a bar graph format. Data represent mean ± SEM (*n* ≥ 3). One-way analysis of variance (ANOVA) was utilized, followed by the Bonferroni post hoc test for pairwise comparisons. *** *p* < 0.001; n.s. = not significant. DMSO: dimethyl sulfoxide.

RANKL significantly enhanced the phosphorylation of key MAPKs, including extracellular signal-regulated kinase (ERK), c-Jun N-terminal kinase (JNK) and p38. EN effectively inhibited the RANKL-induced phosphorylation of these MAPKs (Figure 5). Additionally, EN significantly reduced the RANKL-induced protein levels of c-Fos and NFATc1, important transcription factors in the downstream RANKL pathway (Figure 6). No inhibitory effect of EN was observed on the phosphorylation of NF-κB, another transcription factor.

#### 2.1.5. EN Mitigates H_2_O_2_-Induced Cell Death in Saos-2 Cells

**Figure 7:**
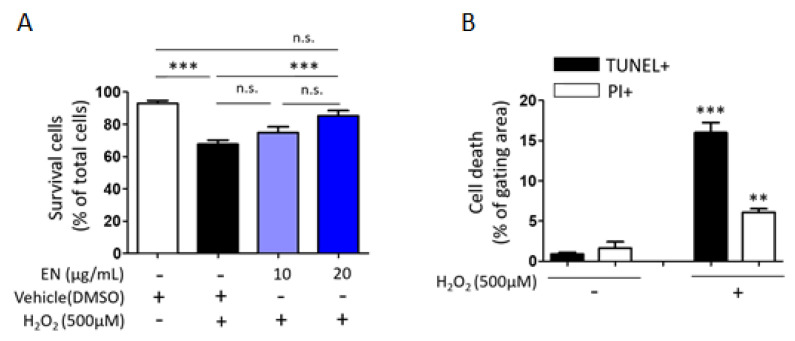
**Protective effect of EN against H_2_O_2_-induced death in Saos-2 cells.** (**A**) H_2_O_2_-induced cell death was assessed using trypan blue exclusion to measure the cell viability. (**B**) Apoptosis or necrosis were quantitatively analyzed in Saos2 cells utilizing TUNEL-PI assays, with outcomes depicted in a bar graph format. Data represent mean ± SEM (*n* ≥ 3). For the comparison of more than three groups (**A**), one-way analysis of variance (ANOVA) was utilized, followed by the Bonferroni post hoc test for pairwise comparisons. ** *p* < 0.01; *** *p* < 0.001; n.s. = not significant. For comparison between two groups (**B**), Student’s *t*-test was employed. ** *p* < 0.01 versus white color bar and *** *p* < 0.001 versus black color bar. DMSO: dimethyl sulfoxide.

**Figure 8:**
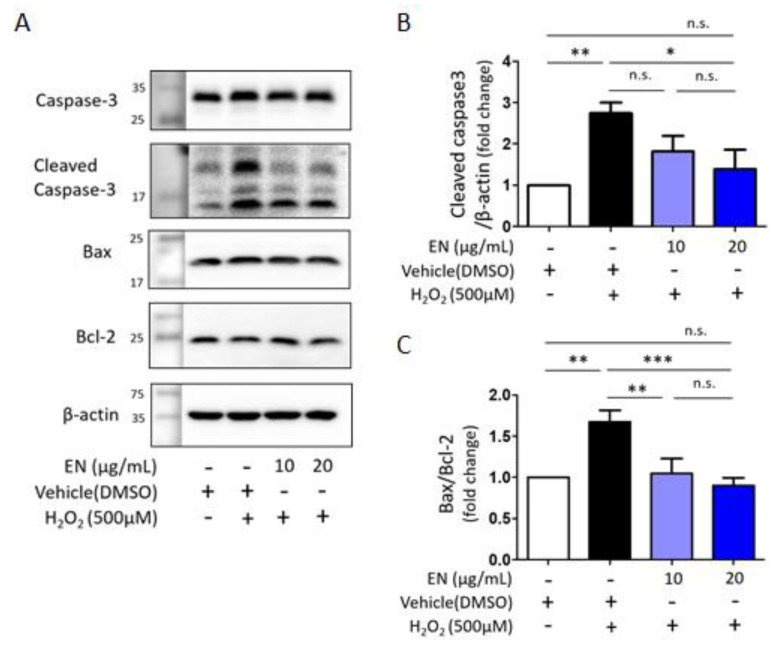
**Expression of apoptotic markers in H_2_O_2_-induced Saos-2 cells.** (**A**) Western blot analysis was used to measure the expression levels of Bax, Bcl-2, caspase-3, and cleaved caspase-3 in the presence of H_2_O_2_ (500 µM) with or without EN (10 and 20 μg/mL) for 16 h. β-actin served as control. (**B**,**C**) The quantified western blot results were displayed as a bar graph, accompanied by statistical analysis. Data represent mean ± SEM (*n* ≥ 3). One-way analysis of variance (ANOVA) was utilized, followed by the Bonferroni post hoc test for pairwise comparisons. * *p* < 0.05; ** *p* < 0.01; *** *p* < 0.001; n.s. = not significant. DMSO: dimethyl sulfoxide.

Considering that osteoblast death is a primary cause of osteoporosis, the ability of EN to regulate osteoblast cell death was investigated. Cells were exposed to hydrogen peroxide (H_2_O_2_), a known inducer of oxidative stress, to trigger cell death in Saos-2 cells. Trypan blue cell counting showed that approximately 32.3% of cells died following H_2_O_2_ treatment (500 µM). However, increasing the concentration of EN (10, 20 μg/mL) significantly reduced cell death to 25.1% and 14.8%, respectively (Figure 7A). To ascertain the predominant type of cell death under these conditions [34], TUNEL-PI assays were conducted, revealing that H_2_O_2_ induced about 15% of TUNEL-positive cell death and 4.5% of PI-positive cell death, compared to the untreated control (0.9% and 1.6%, respectively) (Figure 7B). The mechanisms underlying the protective effect against H_2_O_2_-induced apoptotic cell death were further explored by examining signaling molecules such as caspase-3, Bax, and Bcl-2. EN treatment significantly attenuated the H_2_O_2_-induced caspase-3 activation and Bax/Bcl-2 expression ratio (Figure 8).

## 3. Discussion

When bone resorption increases excessively, bone marrow stromal cells or osteoblasts express and secrete osteoprotegerin (OPG), which interrupts RANKL–RANK binding to maintain bone homeostasis [35,36]. Although the RANKL/RANK/OPG system serves a regulator of bone homeostasis under physiological conditions, OPG’s effectiveness as a decoy receptor for RANKL is compromised in postmenopausal women due to estrogen deficiency. Estrogen has been shown to promote OPG expression and suppress NF-κB activity, thereby inhibiting osteoclast formation and activation [37]. Furthermore, estrogen plays an essential role in osteoblast differentiation and protection [38]. Estrogen deficiency often causes an increase in reactive oxidative stress (ROS) and inflammatory cytokines, which are associated with osteoporosis development [39]. Consequently, the hallmark of osteoporosis in postmenopausal women is characterized by excessive osteoclast activation and osteoblast dysfunction [40]. Our study demonstrates that the ethanol extract of *Neorhodomela munita* (EN) has a protective effect on major bone cells. Specifically, EN attenuated RANKL-induced osteoclast formation and activation by downregulating the MAPKs-NFATc1/c-Fos signaling pathway and reduced H_2_O_2_-induced cell death by decreasing apoptotic signaling, such as caspase 3 activation and the Bax2/Bcl-2 expression ratio. We also investigated caspase 8 activation, which EN did not affect.

Although the compounds isolated from *Neorhodomela munita* are not well understood, Park et al. discovered that polybromocatechols, identified through the reversed-phase HPLC analysis of methanol extracts, exhibited anti-rhinovirus activity in HeLa cell lines [31]. Lim et al. speculated that bromophenols, commonly found in red algae, could demonstrate antioxidant and anti-inflammatory activities [31,32]. We also investigated candidate active compounds of EN using liquid chromatography–mass spectrometry (LC/MS) analysis (Table 1 and Figures S1 and S2). Yajun et al. identified betaine as a candidate with anti-osteoclastogenesis effects in RANKL-induced bone marrow monocyte cells and Raw264.7 cells [41]. Betain has also been noted for its ability to enhance osteoblast differentiation [42]. Choline intake has been suggested to improve bone health, particularly in osteoporosis [43,44]. However, our experiments did not examine the inhibitory effects of these two compounds. We then selected and tested four compounds based on their LC/MS profile with ≥90% match and ≥70% confidence to the mzCloud spectral library (Table S1) [45]. Among these, TRAP staining indicated that monoolein and erucamide had a modest protective effect on RANKL-stimulated Raw264.7 cells at concentrations above 40 μΜ, with the combined treatment of erucamide (20 μΜ) and monoolein (40 μΜ) appearing more effective than erucamide alone (Figure S3). However, quantitative LC/MS analysis revealed that the actual concentrations of these compounds in EN (20 μg/mL) are approximately 0.07 μg/mL (0.2 μM) for monoolein and 0.19 μg/mL (0.7 μM) for eucamide, suggesting they may not be the primary active ingredients.

Various natural compounds have been suggested to block the RANKL–RANK interaction in osteoclastogenesis. For instance, niloticin and ellagic acid were shown to suppress osteoclastogenesis by blocking the RANKL–RANK interaction and inhibiting RANK-mediated signaling pathways such as AKT, MAPK, and NF-κB. In detail, both 2.5 μM of niloticin and 4 μM of ellagic acid reduced TRAP-positive stained cells by around 50% in 50 ng/mL of RANKL-induced Raw264.7 cells [46,47]. Our experiments showed that 20 μg/mL of EN attenuated TRAP-positive stained cells by around 90%, but 10 μg/mL of EN treatment did not reduce TRAP-positive stained cells (Figure 2B). Although our experiments demonstrated that EN could attenuate TRAP-positive stained cells and the activation of RANK-downstream signaling pathways, we were unable to assess EN’s potential to disrupt RANK–RANKL interaction directly, as the main active compounds could not be identified in this study.

We also found that EN could attenuate H_2_O_2_-induced cell death by inhibiting the activation of apoptotic signaling molecules. The protective effects of *Neorhodomela munita* have been observed in cell types such as microglial cells, where methanolic extracts exhibit free radical-scavenging activities, demonstrating neuroprotective effects [32]. Furthermore, extracts and bioactive components derived from various species of red algae have shown antioxidant potential. For example, extracts from *Callophyllis japonica* and *Gracilaria tenuistipitata* have been found to suppress H_2_O_2_-induced cell death and DNA damage, respectively [48,49]. However, it is still difficult to directly compare their antioxidant properties with EN since experiments were performed using the Chinese hamster lung fibroblast line V79-4 and lung cancer cell line. Instead of estimating anti-oxidant ability, we estimated the mitochondrial Bcl-2 expression level (Figure 8). It has been reported that Bcl-2 has antioxidant effector molecules that can reduce intracellular ROS levels, as well as cell survival [50,51]. We further investigated one of the representative apoptotic molecules related to mitochondrial dysfunction, such as Bax, cytochrome C, and caspase 3 (Figure 7). In addition, Akt-GSK3β activation was investigated as another important signaling pathway and used to study oxidative-stress-induced osteoblast cell death in other research groups. Dai et al. examined that Curcumin, a natural antioxidant, has a highly protective effect against H_2_O_2_-induced Saos-2 cell apoptosis by activating Akt-GSK3β signaling [52]. Han et al. demonstrated that chlorogenic acid, known to have a potential antioxidant effect, reduced cell death by reducing oxidative stress via the PI3K/Akt-mediated activation of the Nrf2/HO-1 pathway in MC3T3-E1 cells [53]. Then, the protective effect of EN on H_2_O_2_-induced cell death observed in this study may be due attributed to its antioxidative properties, although further investigation is necessary.

Further research will focus on identifying the active compounds within EN through the fractionation of the crude extracts and an assessment of their pharmacological efficacy. Of particular interest is the exploration of the pharmacokinetics of EN’s active compounds, considering that some current osteoporosis medications have been associated with unexpected long-term release and accumulation in bone tissue, even after the cessation of treatment [54,55].

## 4. Materials and Methods

### 4.1. Preparation of Neorhodomela munita Extract and Its Compounds

The 70% ethanol extract of *Neorhodomela munita* (MABIK NP30210062) was acquired from the National Marine Biodiversity Institute of Korea (MABIK). *N. munita* specimens were collected in June 2021 from Taean-gun, Chungcheongnam-do Province, Korea, under the auspices of MABIK. The freeze-dried whole seaweed of *N. munita* (40 g) underwent extraction through sonication at 40 KHz for 1 h using 70% ethanol (EtOH; 400 mL × 3) at room temperature. Subsequently, the solution was evaporated under reduced pressure (invacuo) after heating under reflux. The yield of the extract [(weight of dry extract (g))/(weight of dry sample (g)) × 100] was 6.25% (*w*/*w*). The resultant dried extract of 70% EtOH *N. munita* (2.5 g dry weight) was preserved at −80 °C pending assessment of its biological activity. Prior to in vitro experiments, the extract was dissolved in dimethyl sulfoxide (DMSO).

### 4.2. The Liquid Chromatography–Mass Spectrometry (LC/MS) Analysis

The extract of *Neorhodomela munita* prepared using 100 ppm in methanol (HPLC grade, Sigma-Aldrich, Saint Louis, MO, USA) was analyzed using a Thermo Q-Exactive Orbitrap plus mass spectrometer connected to a Dionex Ultimate 3000 RSLC nano HPLC using an acquity UPLC BEH C18 (1.7 μm, 2.1 × 100 mm) column (Thermo Fisher Scientific, Waltham, MA, USA). The mobile phase consisted of (A) 0.1% formic acid in HPLC-grade H_2_O and (B) 0.1% formic acid in HPLC-grade acetonitrile at a flow rate of 400 μL/min, with a column temperature of 45 °C. The Orbitrap MS scan was performed in positive ion mode with a 70,000 resolution and a scan range of 80–1000 *m*/*z*. The data-dependent MS/MS was performed with a resolution of 17,500, an isolation window of 2.0 *m*/*z*, and a loop count of 5. The estimation of the elemental composition of ions was performed based on the registered Chemspider (http://www.chemspider.com by Royal Society of Chemistry, London, UK) database and mz Cloud MS database (http://www.mzcloud.org by HighChem LLC, Bratislava, Slovakia).

### 4.3. Cell Culture

The Raw264.7 macrophage cell line (TIB-71; ATCC, Manassas, VA, USA) was maintained in Dulbecco’s modified Eagle’s medium (DMEM) (30-2002; ATCC), supplemented with 10% fetal bovine serum (FBS) (16000-044, Thermo Fisher Scientific), 100 U/mL of penicillin (15140-122, Thermo Fisher Scientific), and 100 μg/mL of streptomycin (15140-122; Thermo Fisher Scientific). The cultures were kept at 37 °C in a humidified incubator under 5% CO_2_ atmosphere. Cells from passages 5 to 15 were utilized for differentiation and treatment experiments. For osteoclast differentiation, Raw264.7 cells were cultured in complete alpha modified minimal essential medium (α-MEM; 11900-024, Thermo Fisher Scientific) supplemented with 40 ng/mL of RANKL (ALX-522-131; ENZO Life Science, Farmingdale, NY, USA), with media changes every 2 days over a period of 4 days.

The human osteoblast-like Saos-2 cell line (KCLB30085; Korean Cell Line Bank, Seoul, Korea) was cultured in RPMI 1640 medium (22400-08, Thermo Fisher Scientific) containing 10% FBS, 100 U/mL of penicillin and 100 μg/mL of streptomycin. These cells were also incubated in a 37 °C humidified incubator with a 5% CO_2_ atmosphere.

### 4.4. Evaluation of Cell Viability (WST-1)

Cell viability was assessed using the water-soluble tetrazolium 1 (WST-1) assay. In particular, the EZ-Cytox water-soluble tetrazolium salt (WST) assay kit (EZ-3000; Dogenbio, Seoul, Republic of Korea) was employed, following the manufacturer’s instructions. Absorbance was measured at 450 nm using a microplate reader (Multiskan FC; Thermo Fisher Scientific).

### 4.5. Reverse Transcription PCR (RT-PCR)

Total RNA was isolated from cells using the Hybrid-R-kit (305-101; GeneAll Biotechnology, Seoul, Republic of Korea). For cDNA synthesis, 1 μg of total RNA was reverse transcribed using the Maxime RT PreMix Kit (25081; iNtRON Biotechnology, Seongnam, Republic of Korea), following the manufacturer’s instructions. RT-PCR was carried out in a PCR machine (C1000 touch Thermal cycler; Bio-Rad, Hercules, CA, USA) using AccuPower^®^ PCR PreMix (K-2016; Bioneer, Daejeon, Republic of Korea). The cycling conditions were as follows: 95 °C for 20 s for denaturation, 56 °C for 30 s for annealing, and 72 °C for 30 s for extension across 35 cycles, with final extension at 72 °C for 5 min. RT-PCR was performed in triplicate or more for each samples. The RT-PCR products were then electrophoresed on a 1.2–1.5% agarose gel, and the gel was visualized using a BioRad ChemiDoc XRS imaging system (Bio-Rad); the gel images were analyzed with NIH ImageJ 1.52a software. The relative gene expression was normalized to Glyceraldehyde 3-phosphate dehydrogenase (GAPDH). All primer pairs were synthesized using Bioneer.

The mouse (m)-specific primers used are as follows: (m) Cathepsin K (forward: 5′-GCA GAT GTT TGT GTT GGT CTC T-3′; reverse: 5′-TGG TGG AAA GGT GTG ACA GG-3′), (m)DC-STAMP (forward: 5′-TTG AAC CGA GCT GCA TTC CT-3′; reverse: 5′-GCA CTA CCT TGG CCT TAC CT-3′), (m)NFATc1 (forward: 5′-GGA GAG TCC GAG AAT CGA GAT-3′; reverse: 5′-TTG CAG CTA GGA AGT ACG TCT-3′), (m)TRAP (forward: 5′-CTC CTG CCT GTT CTC TTC CCA-3′; reverse: 5′-AAG AGA GAA AGT CAA GGG AGT GGC-3′), (m)OSCAR (forward: 5′-CCC AGT CTG TCT TGC GGT AG-3′; reverse: 5′-TCT GGG TTG GAG GGT CCT AA-3′), (m)GAPDH (forward: 5′-CAA GGT CAT CCA TGG ACA ACT TTG-3′; reverse: 5′-GTC CAC CAC CCT GTT GCT GTA G-3′).

### 4.6. Western Blot

To prepare protein samples, the cells were lysed in RIPA buffer (25 mM of Tris pH 7.6, 150 mM of NaCl, 1% NP-40, 1% sodium deoxycholate, 0.1% sodium dodecyl sulfate (SDS)) supplemented with a protease inhibitor (sc-11697498001; Santa Cruz Biotechnology, Inc., Dallas, TX, USA) and phosphatase inhibitors (4906845001; Thermo Fisher Scientific). The protein concentration was determined using the Bicinchoninic Acid (BCA) assay. A 12% SDS-polyacrylamide gel was used to separate samples, which were then transferred onto immobilon-P PVDF membranes (IPVH00010; Merk Millipore, Burlington, MA, USA). The membranes were blocked with 5% skim milk for 30 min at room temperature, and incubated overnight at 4 °C with primary antibodies. Following this, the membranes were incubated with secondary antibodies diluted in 5% skim milk in Tris-buffered saline (TBS) containing 0.1% Tween-20 (TBST) for 1 h at room temperature. The protein bands were visualized using enhanced chemiluminescence (ECL) reagent (AbClon, Seoul, Republic of Korea) and quantified with ImageJ 1.52a software.

Primary and secondary antibodies were used at the following dilutions: Primary antibodies; ERK (1:1000; Cell signaling Technology, Danver, MA, USA, 9102), phospho-ERK (1:1000; Santa Cruz, sc-7383), p38 (1:1000; Cell signaling, 9212), phospho-p38 (1:1000; Cell signaling, 9211), JNK (1:1000; Cell signaling, 9252), phospho-JNK (1:1000; Cell signaling, 9251), NFATc1 (1:1000; Santa Cruz, sc7294), c-fos (1:1000; Santa Cruz, sc-166940), Bax (1:1000; Abcam, ab32503), Bcl-2 (1:1000; Santa Cruz, sc7382), Caspase3 (1:1000; Cell signaling, 9662), Beta actin (1:5000; Santa Cruz, sc47778). Secondary antibodies; anti-mouse (1:2000; ENZO, ADI-SAB-100J) or anti-rabbit (1:2000; ENZO, ADI-SAB-300-J).

### 4.7. TRAP Staining

Tartrate-Resistant Acid Phosphatase (TRAP) staining was conducted using the TRACP and ALP double-stain kit (MK300; TAKARA), following the manufacturer’s protocol. Briefly, Raw264.7 cells were seeded into a 48-well culture plate at a density of 4 × 10^3^ cells per well in DMEM medium. Once adherent, the cells were treated with DMSO (as a vehicle control) and varying concentrations of Neorhodomela aculeate extract (EN) (10 μg/mL and 20 μg/mL) in α-MEM medium for 1 h. Then, RANKL (40 ng/mL) and EN were administered every 2 days over a 4-day period. Following adequate differentiation, cells were gently rinsed with PBS and fixed with 120 μL of fixation solution for 5 min at room temperature. Subsequently, 120 μL of substrate solution was applied to each well and incubated at 37 °C for 45 min. The solution was then aspirated, and the cells were mounted with glycerol to prevent drying. TRAP-positive cells were identified and imaged using a microscope (CKX41; Olympus, Tokyo, Japan) equipped with a digital camera (eXcope T300; Olympus). Osteoclasts staining purple were counted as TRAP-positive cells across three or more independent experiments. The number of TRAP-positive cells was quantified using NIH ImageJ 1.52a software (Silk Scientific Corp., Orem, UT, USA).

### 4.8. Bone Resorption Assay

The bone resorption assay was conducted using a bone resorption assay kit (CSR-BRA-48KIT; COSMOBIO, Tokyo, Japan), following the manufacturer’s instructions with slight modifications. For this assay, Raw264.7 cells (4 × 10^3^ cells/well) were seeded onto a calcium phosphate (CaP) and collagen-type I-coated 48-well culture plate. A 50 μg/mL coating of collagen type I (3447-020-01; R&D System, Minneapolis, MN, USA) was applied to create a bone biomimetic surface. The cells were then cultured at 37 °C with 5% CO_2_ in DMEM supplemented with 10% FBS. After 24 h, the cells were pre-treated with DMSO (vehicle) and varying concentrations of EN (10 μg/mL and 20 μg/mL) in α-MEM medium for 1 h. RANKL (100 ng/mL) was added to the medium to induce osteoclast differentiation over a period of 7 days. At the end of the culture period, cells were rinsed with PBS and treated with 5% sodium hypochlorite to detach the cells. Following this, the plate was washed with distilled water (D.W.) and allowed to dry. The area of bone resorption was imaged using a digital camera (Olympus) attached to a microscope (Olympus). The extent of the resorptive pits was quantified using NIH ImageJ 1.52a software.

### 4.9. Actin Ring Formation

To visualize the actin ring formation in osteoclasts differentiated from Raw264.7 cells, staining with Texas Red™-X Phalloidin (T7471; Thermo Fisher Scientific) was performed. Initially, Raw264.7 cells (1 × 10^4^ cells/well) were seeded onto a 4-well chamber slide. Following treatment with RANKL with or without EN (20 μg/mL) for 4 days, the cells were rinsed with PBS and fixed with 4% paraformaldehyde in PBS for 10 min at room temperature. The cells were then permeabilized with 0.1% triton X-100 in PBS for 5 min at room temperature. After a PBS wash, cells were blocked with 1% BSA for 30 min at room temperature. Post blocking, cells were stained with 2.5% Texas Red™-X Phalloidin in PBS for 20 min at room temperature. Additionally, cell nuclei were stained with diamidino-2-pehnylindole (DAPI) (1:1000; D21490; Thermo Fisher Scientific) in a dark environment for 5 min. The visualization of osteoclast actin ring formation was achieved using a LSM 700 laser scanning confocal microscope (Carl zeiss, Overkochen, Germany).

### 4.10. Osteoblast Apoptosis Induced by H_2_O_2_

The treatment of osteoblast-like Saos-2 cells with H_2_O_2_ was carried out as follows: Saos-2 cells were plated in a 60 mm culture dish at a density of 2 × 10^5^ cells per well using complete RPMI 1640 medium (Thermo Fisher Scientific). Upon reaching approximately 70% confluence, the cells were exposed to 500 μM of H_2_O_2_ for 16 h to induce cell death. Subsequent to the H_2_O_2_ treatment, both the supernatant and cells were collected and processed for further experimental analysis.

### 4.11. Cell Counting Assay (Trypan Blue Viability Assay)

The protective effect of *Neorhodomela munita* extract (EN) against H_2_O_2_-induced cytotoxicity in saos-2 cells was assessed using a cell-counting method. Saos-2 cells were cultured at 37 °C in an atmosphere containing 5% CO_2_ in complete RPMI 1640 medium. The cells were pre-treated with DMSO (as a vehicle control) and various concentrations of EN (10 and 20 μg/mL) for 1 h, followed by exposure to 500 μM of H_2_O_2_ for 16 h. Post treatment, both the attached cells and supernatant were collected and centrifuged. An equal volume of the cell suspension was then mixed with trypan blue stain to assess the cell viability. This process was replicated in three or more independent experiments, with viability measurements taken using the NanoEnTek Eve Automated Cell Counter (NanoEnTek, Seoul, Republic of Korea).

### 4.12. TUNEL-Propidium Iodide (PI) Assay

The In situ Direct DNA Fragmentation (TUNEL) assay kit (ab66108; Abcam, Waltham, MA, USA) was employed to identify apoptotic cells, following the manufacturer’s instructions. Initially, treated cells and the centrifuged supernatant were fixed with 1% paraformaldehyde in PBS and placed on ice for 15 min, followed by a gentle rinse with PBS. Subsequently, the cells were treated with 5 mL of ice-cold 70% ethanol in D.W. and stored at −20 °C until further analysis. The fixed cells were then re-suspended in a wash buffer and stained with DNA labeling solution at 37 °C for 1 h. Prior to the addition of the PI/RNase A solution, the cells underwent two wash cycles with rinse buffer. The final step involved incubating the cells in the dark for 30 min at room temperature. The assessment of apoptosis and necrosis was performed using BD Accuri^TM^ C6 Plus flow cytometry (BD Biosciences, Mississauga, ON, Canada) and analyzed with BD Accuri^TM^ C6 Plus Software (BD Biosciences) (Ex/Em = 488/520 nm for FITC, and 488/623 nm for PI).

### 4.13. Statistical Analysis

Statistical analyses of all data were performed using GraphPad Prism 5 software (GraphPad Software, San Diego, CA, USA). Experiments were conducted more than three times to ensure reproducibility. Results are presented as mean ± standard error of the mean (SEM). For comparison between two groups, the two-tailed Student’s *t*-test was employed. When more than three groups were analyzed, one-way analysis of variance (ANOVA) was utilized, followed by the Bonferroni post hoc test for pairwise comparisons. A *p*-value of less than 0.05 was considered to indicate statistical significance.

## 5. Conclusions

This study uncovered a novel function of the 70% ethanol extract of *Neorhodomela munita* (EN). We demonstrate its protective effects against both excessive osteoclastogenesis and osteoblast apoptosis, key phenomena in the pathogenesis of osteoporosis; EN showed its anti-osteoclastic effect at both 10 and 20 μg/mL and its anti-apoptotic effect at 20 μg/mL in vitro. While our findings highlight the potential benefits of EN for osteoporosis treatment based on in vitro studies with a crude extract, further detailed investigations are required for this to be advanced to in vivo preclinical and clinical testing phases. Ultimately, this study lays the groundwork for the development of a novel pharmaceutical candidate for osteoporosis, promising new avenues for treatment strategies.

## Figures and Tables

**Table 1:** LC/MS profile of the 70% ethanol *Neorhodomela munita* extract (EN).

No.	RT (Min.)	Formula	Candidate M.W.	Area (Max.)	Name by Searching ChemSpider Results
1	0.762	C_6_ H_13_ N O_2_	131.0945	341,181,829.574	6-Aminocaproic acid
2	7.26	C_11_ H_16_ O_3_	196.1097	187,512,214.227	1-carboxy-3-hydroxyadamantane
3	0.705	C_5_ H_13_ N O	103.0998	181,541,412.769	Choline
4	6.522	C_6_ H_18_ O_3_ Si_3_	222.0556	141,499,620.568	Hexamethylcyclotrisiloxane
5	7.431	C_11_ H_16_ O_3_	196.1097	117,850,468.790	1-carboxy-3-hydroxyadamantane
6	0.814	C_5_ H_11_ N O_2_	117.0789	112,852,646.944	Betaine
7	6.6	C_10_ H_12_ Br N O_4_	288.9944	112,762,617.285	Amino(5-bromo-2,4-dimethoxyphenyl)acetic acid
8	0.785	C_5_ H_9_ N O_2_	115.0633	93,627,111.482	D-(+)-Proline
9	8.553	C_11_ H_16_ O_2_	180.1148	74,265,194.775	2-tert-Butyl-4-methoxyphenol
10	1.25	C_6_ H_6_ N_2_ O	122.048	62,495,868.421	Nicotinamide

## Data Availability

All data are available in this publication.

## Supplementary Materials

The following supporting information can be downloaded at: https://www.mdpi.com/article/10.3390/molecules29081741/s1, Table S1. LC/MS profile of 4 compounds with ≥90% match to the mzCloud spectral library, Figure S1: LC/MS chromatograms of a 70% ethanol *Neorhodomela munita* extract (EN), Figure S2: LC/MS analysis of a 70% ethanol *Neorhodomela munita* extract (EN), Figure S3: Four candidate compounds were further screened on their anti-osteoclastic effect in vitro.

## References

[1] Sarafrazi N., Wambogo E.A., Shepherd J.A. (2021). Osteoporosis or Low Bone Mass in Older Adults: United States, 2017–2018. NCHS Data Brief.

[2] Salari N., Darvishi N., Bartina Y., Larti M., Kiaei A., Hemmati M., Shohaimi S., Mohammadi M. (2021). Global prevalence of osteoporosis among the world older adults: A comprehensive systematic review and meta-analysis. J. Orthop. Surg. Res..

[3] GBD 2019 Fracture Collaborators (2021). Global, regional, and national burden of bone fractures in 204 countries and territories, 1990–2019: A systematic analysis from the Global Burden of Disease Study 2019. Lancet Healthy Longev..

[4] LeBoff M.S., Greenspan S.L., Insogna K.L., Lewiecki E.M., Saag K.G., Singer A.J., Siris E.S. (2022). The clinician’s guide to prevention and treatment of osteoporosis. Osteoporos. Int..

[5] Rashki Kemmak A., Rezapour A., Jahangiri R., Nikjoo S., Farabi H., Soleimanpour S. (2020). Economic burden of osteoporosis in the world: A systematic review. Med. J. Islam. Repub. Iran..

[6] Shen Y., Huang X., Wu J., Lin X., Zhou X., Zhu Z., Pan X., Xu J., Qiao J., Zhang T. (2022). The Global Burden of Osteoporosis, Low Bone Mass, and Its Related Fracture in 204 Countries and Territories, 1990–2019. Front. Endocrinol..

[7] Moayyeri A., Warden J., Han S., Suh H.S., Pinedo-Villanueva R., Harvey N.C., Curtis J.R., Silverman S., Multani J.K., Yeh E.J. (2023). Estimating the economic burden of osteoporotic fractures in a multinational study: A real-world data perspective. Osteoporos. Int..

[8] Ginaldi L., Di Benedetto M.C., De Martinis M. (2005). Osteoporosis, inflammation and ageing. Immun. Ageing.

[9] Xu J., Yu L., Liu F., Wan L., Deng Z. (2023). The effect of cytokines on osteoblasts and osteoclasts in bone remodeling in osteoporosis: A review. Front. Immunol..

[10] Zha L., He L., Liang Y., Qin H., Yu B., Chang L., Xue L. (2018). TNF-alpha contributes to postmenopausal osteoporosis by synergistically promoting RANKL-induced osteoclast formation. Biomed. Pharmacother..

[11] Feng W., Liu H., Luo T., Liu D., Du J., Sun J., Wang W., Han X., Yang K., Guo J. (2022). Author Correction: Combination of IL-6 and sIL-6R differentially regulate varying levels of RANKL-induced osteoclastogenesis through NF-kappaB, ERK and JNK signaling pathways. Sci. Rep..

[12] Yasuda H., Shima N., Nakagawa N., Yamaguchi K., Kinosaki M., Mochizuki S., Tomoyasu A., Yano K., Goto M., Murakami A. (1998). Osteoclast differentiation factor is a ligand for osteoprotegerin/osteoclastogenesis-inhibitory factor and is identical to TRANCE/RANKL. Proc. Natl. Acad. Sci. USA.

[13] Park J.H., Lee N.K., Lee S.Y. (2017). Current Understanding of RANK Signaling in Osteoclast Differentiation and Maturation. Mol. Cells.

[14] Zenz R., Eferl R., Scheinecker C., Redlich K., Smolen J., Schonthaler H.B., Kenner L., Tschachler E., Wagner E.F. (2008). Activator protein 1 (Fos/Jun) functions in inflammatory bone and skin disease. Arthritis Res. Ther..

[15] Nedeva I.R., Vitale M., Elson A., Hoyland J.A., Bella J. (2021). Role of OSCAR Signaling in Osteoclastogenesis and Bone Disease. Front. Cell Dev. Biol..

[16] Kim J.H., Kim N. (2014). Regulation of NFATc1 in Osteoclast Differentiation. J. Bone Metab..

[17] Noh J.Y., Yang Y., Jung H. (2020). Molecular Mechanisms and Emerging Therapeutics for Osteoporosis. Int. J. Mol. Sci..

[18] Ukon Y., Makino T., Kodama J., Tsukazaki H., Tateiwa D., Yoshikawa H., Kaito T. (2019). Molecular-Based Treatment Strategies for Osteoporosis: A Literature Review. Int. J. Mol. Sci..

[19] Saag K.G., Emkey R., Schnitzer T.J., Brown J.P., Hawkins F., Goemaere S., Thamsborg G., Liberman U.A., Delmas P.D., Malice M.P. (1998). Alendronate for the prevention and treatment of glucocorticoid-induced osteoporosis. N. Engl. J. Med..

[20] Reid D.M., Devogelaer J.P., Saag K., Roux C., Lau C.S., Reginster J.Y., Papanastasiou P., Ferreira A., Hartl F., Fashola T. (2009). Zoledronic acid and risedronate in the prevention and treatment of glucocorticoid-induced osteoporosis (HORIZON): A multicentre, double-blind, double-dummy, randomised controlled trial. Lancet.

[21] Zhou J., Ma X., Wang T., Zhai S. (2016). Comparative efficacy of bisphosphonates in short-term fracture prevention for primary osteoporosis: A systematic review with network meta-analyses. Osteoporos. Int..

[22] Trovas G.P., Lyritis G.P., Galanos A., Raptou P., Constantelou E. (2002). A randomized trial of nasal spray salmon calcitonin in men with idiopathic osteoporosis: Effects on bone mineral density and bone markers. J. Bone Miner. Res..

[23] Wu L., Ling Z., Feng X., Mao C., Xu Z. (2017). Herb Medicines against Osteoporosis: Active Compounds & Relevant Biological Mechanisms. Curr. Top. Med. Chem..

[24] Abdel-Naim A.B., Alghamdi A.A., Algandaby M.M., Al-Abbasi F.A., Al-Abd A.M., Eid B.G., Abdallah H.M., El-Halawany A.M. (2018). Rutin Isolated from *Chrozophora tinctoria* Enhances Bone Cell Proliferation and Ossification Markers. Oxidative Med. Cell Longev..

[25] Kim S.K., Pangestuti R. (2011). Biological activities and potential health benefits of fucoxanthin derived from marine brown algae. Adv. Food Nutr. Res..

[26] Airanthi M.K., Hosokawa M., Miyashita K. (2011). Comparative antioxidant activity of edible Japanese brown seaweeds. J. Food Sci..

[27] Saadaoui I., Rasheed R., Abdulrahman N., Bounnit T., Cherif M., Al Jabri H., Mraiche F. (2020). Algae-Derived Bioactive Compounds with Anti-Lung Cancer Potential. Mar. Drugs.

[28] Ramos-Romero S., Torrella J.R., Pages T., Viscor G., Torres J.L. (2021). Edible Microalgae and Their Bioactive Compounds in the Prevention and Treatment of Metabolic Alterations. Nutrients.

[29] Carvalhal F., Cristelo R.R., Resende D., Pinto M.M.M., Sousa E., Correia-da-Silva M. (2019). Antithrombotics from the Sea: Polysaccharides and Beyond. Mar. Drugs.

[30] Jiang Z., Li R., Cui Y., Jia X., Liu T., Wang X., Qu J. (2021). The complete mitochondrial genome and phylogenetic analysis of *Neorhodomela munita*. Mitochondrial DNA B Resour..

[31] Park S.H., Song J.H., Kim T., Shin W.S., Park G.M., Lee S., Kim Y.J., Choi P., Kim H., Kim H.S. (2012). Anti-human rhinoviral activity of polybromocatechol compounds isolated from the rhodophyta, *Neorhodomela aculeata*. Mar. Drugs.

[32] Lim C.S., Jin D.Q., Sung J.Y., Lee J.H., Choi H.G., Ha I., Han J.S. (2006). Antioxidant and anti-inflammatory activities of the methanolic extract of *Neorhodomela aculeate* in hippocampal and microglial cells. Biol. Pharm. Bull..

[33] Lakkakorpi P.T., Vaananen H.K. (1991). Kinetics of the osteoclast cytoskeleton during the resorption cycle in vitro. J. Bone Miner. Res..

[34] Teramoto S., Tomita T., Matsui H., Ohga E., Matsuse T., Ouchi Y. (1999). Hydrogen peroxide-induced apoptosis and necrosis in human lung fibroblasts: Protective roles of glutathione. Jpn. J. Pharmacol..

[35] Udagawa N., Takahashi N., Yasuda H., Mizuno A., Itoh K., Ueno Y., Shinki T., Gillespie M.T., Martin T.J., Higashio K. (2000). Osteoprotegerin produced by osteoblasts is an important regulator in osteoclast development and function. Endocrinology.

[36] Brandstrom H., Bjorkman T., Ljunggren O. (2001). Regulation of osteoprotegerin secretion from primary cultures of human bone marrow stromal cells. Biochem. Biophys. Res. Commun..

[37] Cheng C.H., Chen L.R., Chen K.H. (2022). Osteoporosis Due to Hormone Imbalance: An Overview of the Effects of Estrogen Deficiency and Glucocorticoid Overuse on Bone Turnover. Int. J. Mol. Sci..

[38] Almeida M., Iyer S., Martin-Millan M., Bartell S.M., Han L., Ambrogini E., Onal M., Xiong J., Weinstein R.S., Jilka R.L. (2013). Estrogen receptor-alpha signaling in osteoblast progenitors stimulates cortical bone accrual. J. Clin. Investig..

[39] Iantomasi T., Romagnoli C., Palmini G., Donati S., Falsetti I., Miglietta F., Aurilia C., Marini F., Giusti F., Brandi M.L. (2023). Oxidative Stress and Inflammation in Osteoporosis: Molecular Mechanisms Involved and the Relationship with microRNAs. Int. J. Mol. Sci..

[40] (2021). Management of osteoporosis in postmenopausal women: The 2021 position statement of The North American Menopause Society. Menopause.

[41] Yajun W., Jin C., Zhengrong G., Chao F., Yan H., Weizong W., Xiaoqun L., Qirong Z., Huiwen C., Hao Z. (2021). Betaine Attenuates Osteoarthritis by Inhibiting Osteoclastogenesis and Angiogenesis in Subchondral Bone. Front. Pharmacol..

[42] Villa I., Senesi P., Montesano A., Ferraretto A., Vacante F., Spinello A., Bottani M., Bolamperti S., Rubinacci A., Luzi L. (2017). Betaine promotes cell differentiation of human osteoblasts in primary culture. J. Transl. Med..

[43] Oyen J., Gjesdal C.G., Karlsson T., Svingen G.F., Tell G.S., Strand E., Drevon C.A., Vinknes K.J., Meyer K., Ueland P.M. (2017). Dietary Choline Intake Is Directly Associated with Bone Mineral Density in the Hordaland Health Study. J. Nutr..

[44] Spector T.D., Calomme M.R., Anderson S.H., Clement G., Bevan L., Demeester N., Swaminathan R., Jugdaohsingh R., Berghe D.A., Powell J.J. (2008). Choline-stabilized orthosilicic acid supplementation as an adjunct to calcium/vitamin D3 stimulates markers of bone formation in osteopenic females: A randomized, placebo-controlled trial. BMC Musculoskelet. Disord..

[45] Wright Muelas M., Roberts I., Mughal F., O’Hagan S., Day P.J., Kell D.B. (2020). An untargeted metabolomics strategy to measure differences in metabolite uptake and excretion by mammalian cell lines. Metabolomics.

[46] Xu H., Jia Y., Li J., Huang X., Jiang L., Xiang T., Xie Y., Yang X., Liu T., Xiang Z. (2022). Niloticin inhibits osteoclastogenesis by blocking RANKL-RANK interaction and suppressing the AKT, MAPK, and NF-kappaB signaling pathways. Biomed. Pharmacother..

[47] Xu H., Chen F., Liu T., Xu J., Li J., Jiang L., Wang X., Sheng J. (2020). Ellagic acid blocks RANKL-RANK interaction and suppresses RANKL-induced osteoclastogenesis by inhibiting RANK signaling pathways. Chem. Biol. Interact..

[48] Kang K.A., Bu H.D., Park D.S., Go G.M., Jee Y., Shin T., Hyun J.W. (2005). Antioxidant activity of ethanol extract of *Callophyllis japonica*. Phytother. Res..

[49] Yang J.I., Yeh C.C., Lee J.C., Yi S.C., Huang H.W., Tseng C.N., Chang H.W. (2012). Aqueous extracts of the edible *Gracilaria tenuistipitata* are protective against H_2_O_2_-induced DNA damage, growth inhibition, and cell cycle arrest. Molecules.

[50] Kane D.J., Sarafian T.A., Anton R., Hahn H., Gralla E.B., Valentine J.S., Ord T., Bredesen D.E. (1993). Bcl-2 inhibition of neural death: Decreased generation of reactive oxygen species. Science.

[51] Giardino I., Edelstein D., Brownlee M. (1996). BCL-2 expression or antioxidants prevent hyperglycemia-induced formation of intracellular advanced glycation endproducts in bovine endothelial cells. J. Clin. Investig..

[52] Dai P., Mao Y., Sun X., Li X., Muhammad I., Gu W., Zhang D., Zhou Y., Ni Z., Ma J. (2017). Attenuation of Oxidative Stress-Induced Osteoblast Apoptosis by Curcumin is Associated with Preservation of Mitochondrial Functions and Increased Akt-GSK3beta Signaling. Cell Physiol. Biochem..

[53] Han D., Chen W., Gu X., Shan R., Zou J., Liu G., Shahid M., Gao J., Han B. (2017). Cytoprotective effect of chlorogenic acid against hydrogen peroxide-induced oxidative stress in MC3T3-E1 cells through PI3K/Akt-mediated Nrf2/HO-1 signaling pathway. Oncotarget.

[54] Watts N.B., Diab D.L. (2010). Long-term use of bisphosphonates in osteoporosis. J. Clin. Endocrinol. Metab..

[55] Miller P.D. (2011). A review of the efficacy and safety of denosumab in postmenopausal women with osteoporosis. Ther. Adv. Musculoskelet. Dis..

