# Supplementary material for: A 70% Ethanol Neorhodomela munita Extract Attenuates RANKL-Induced Osteoclast Activation and H2O2-Induced Osteoblast Apoptosis In Vitro
Source: Molecules. 2024 Apr 11;29(8):1741. doi: 10.3390/molecules29081741 (PMC11052068; doi:10.3390/molecules29081741)

A 70% ethanol *Neorhodomela munita* extracts attenuates RANKL-induced osteoclast activation and H_2O_2-induced osteoblast apoptosis in vitro

Seongtae Jeong[1,#], Il-Kwon Kim[2,#], Hanbyeol Moon[3], Hojin Kim[4], Byeong-Wook Song[5], Jung-Won Choi[6], Sang Woo Kim[5], Seahyoung Lee[5], Dong-Sik Chae[7,*], Soyeon Lim[5,*]

Supplementary Figures

Figure S1. LC/MS chromatograms of a 70% ethanol *Neorhodomela munita* extract (EN)

Blank

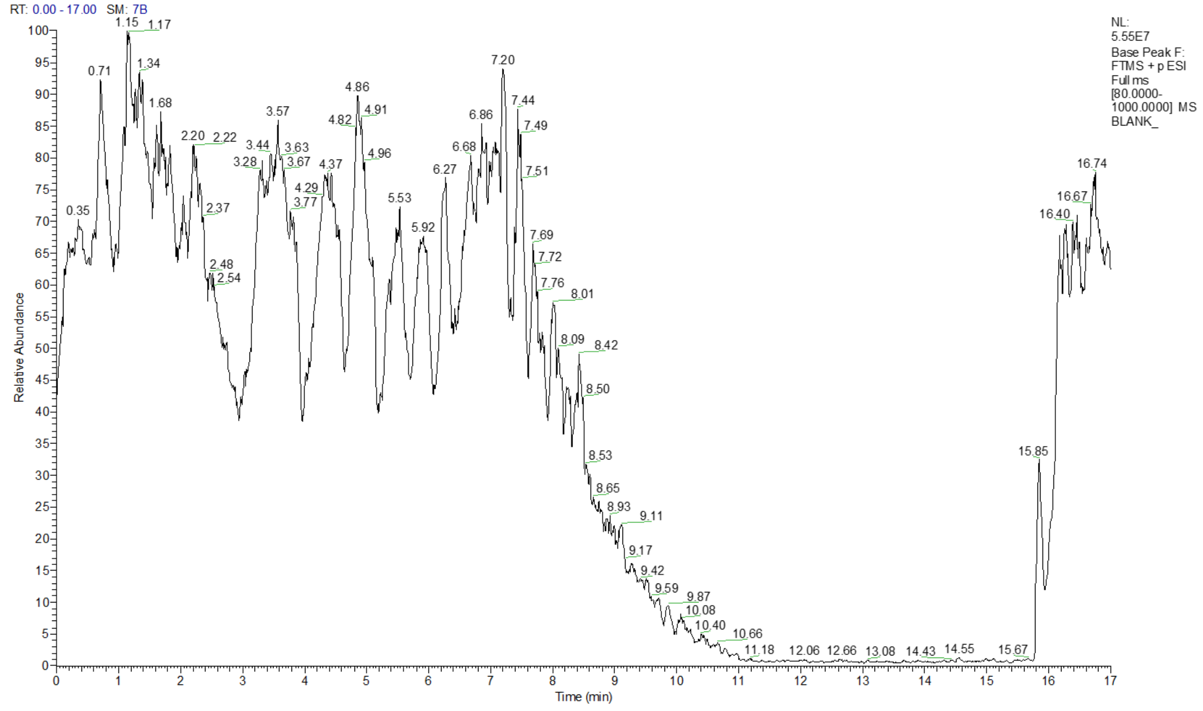

EN

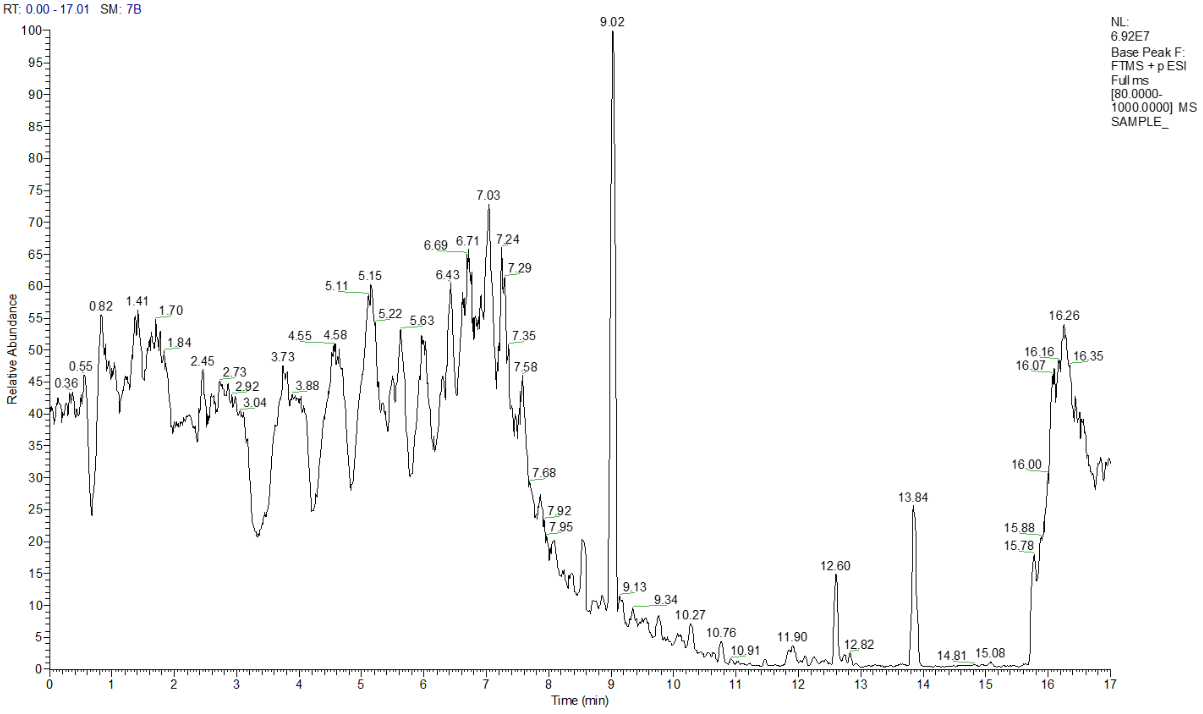

Figure S2. LC/MS analysis of a 70% ethanol *Neorhodomela munita* extract (EN)

No. 1. 6-Aminocaproic acid

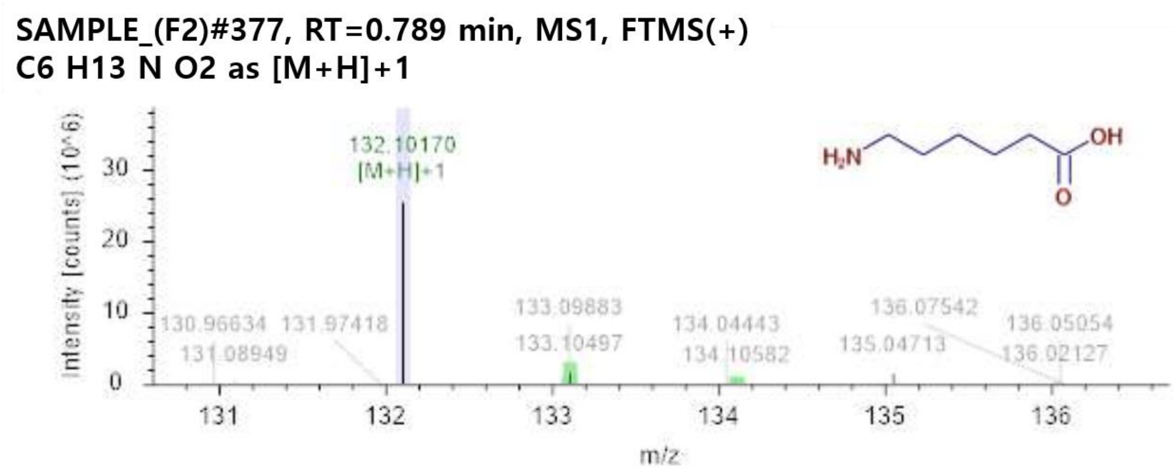

No. 2. 1-carboxy-3-hydroxyadamantane

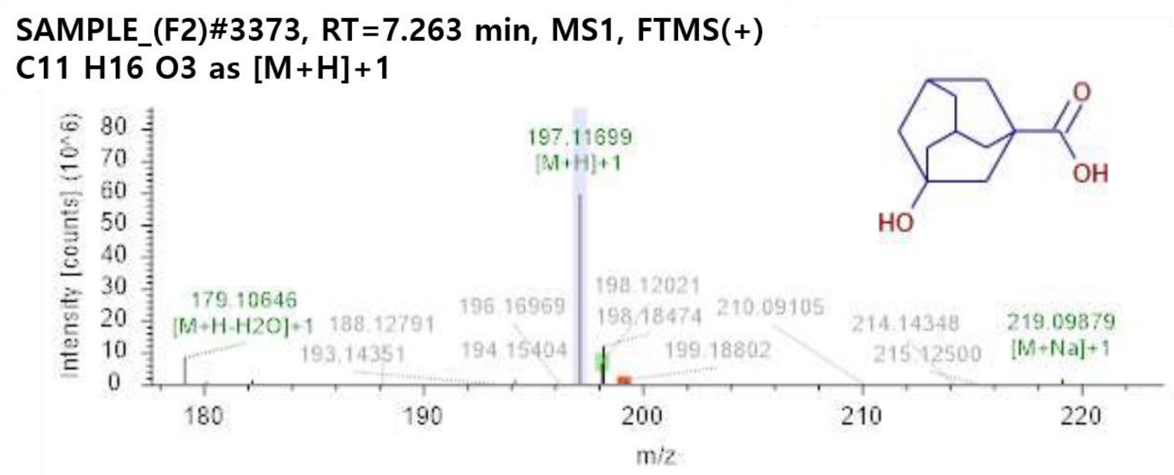

No. 3. Choline

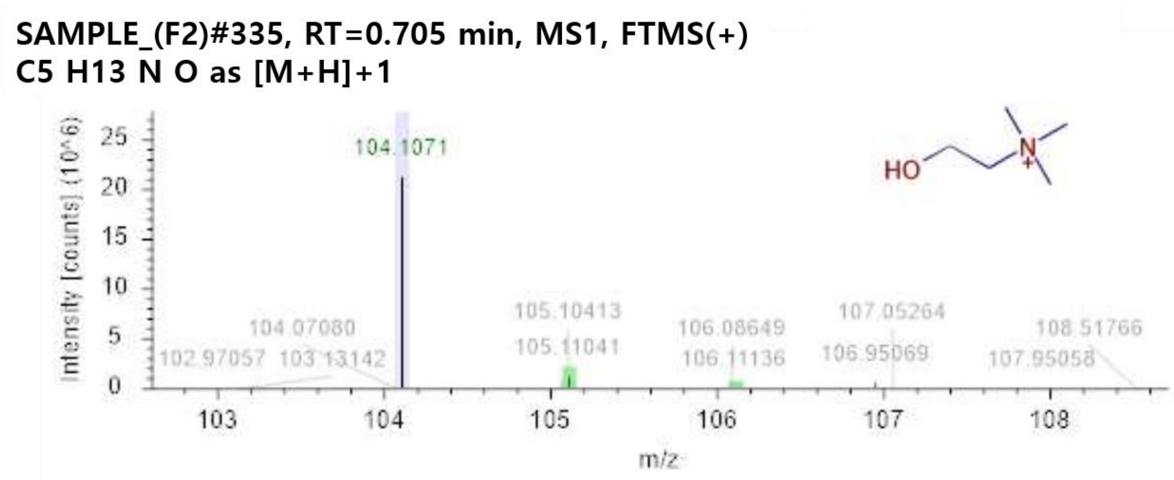

No. 4. Hexamethylcyclotrisiloxane

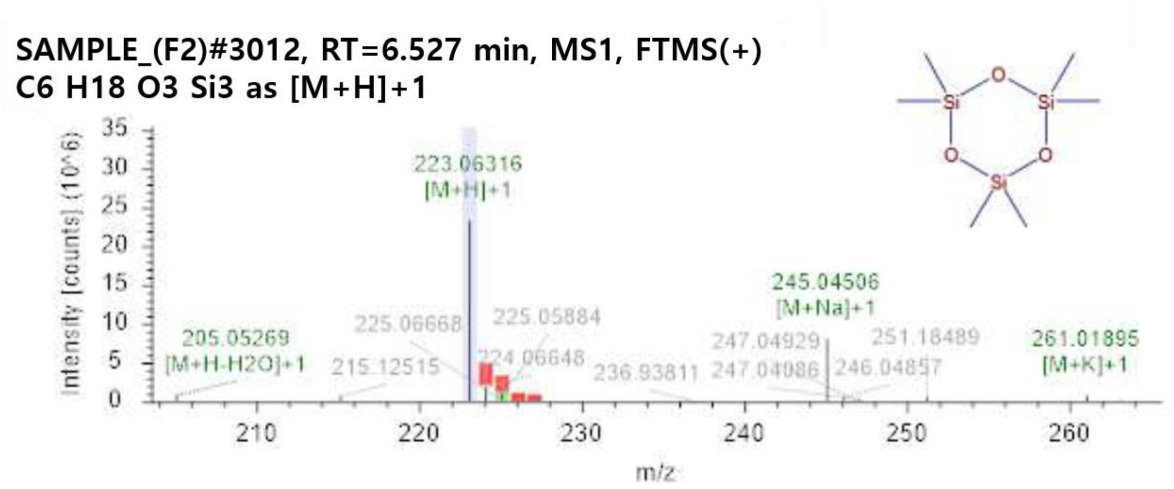

No. 5. 1-carboxy-3-hydroxyadamantane

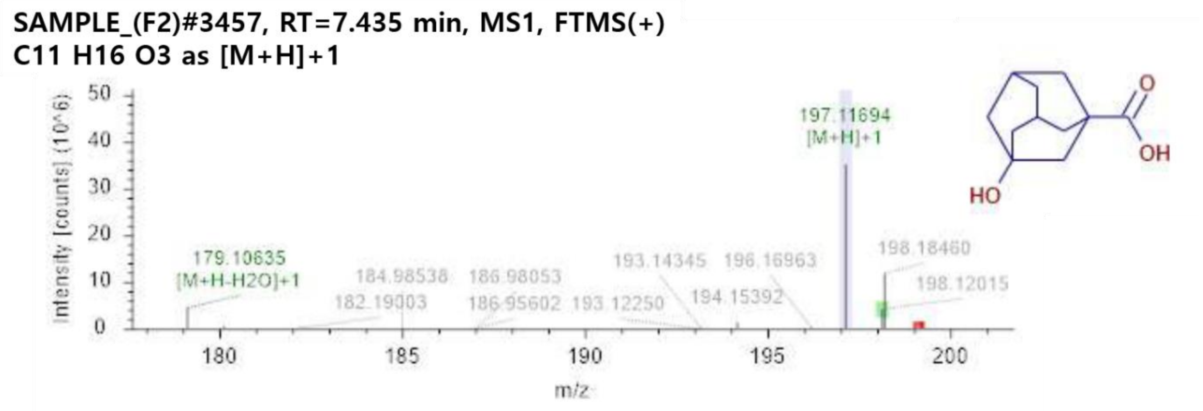

No. 6. Betaine

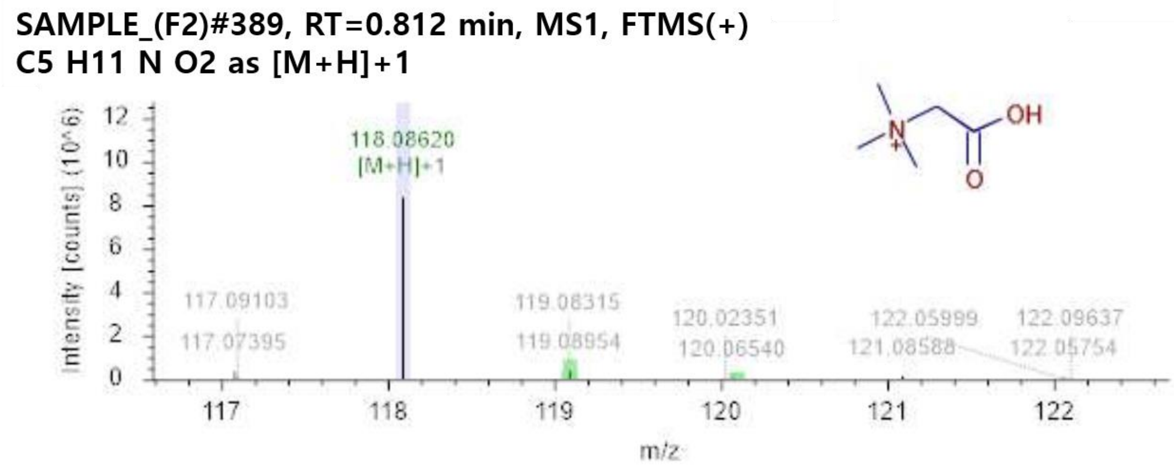

No. 7. Amino(5-bromo-2,4-dimethoxyphenyl)acetic acid

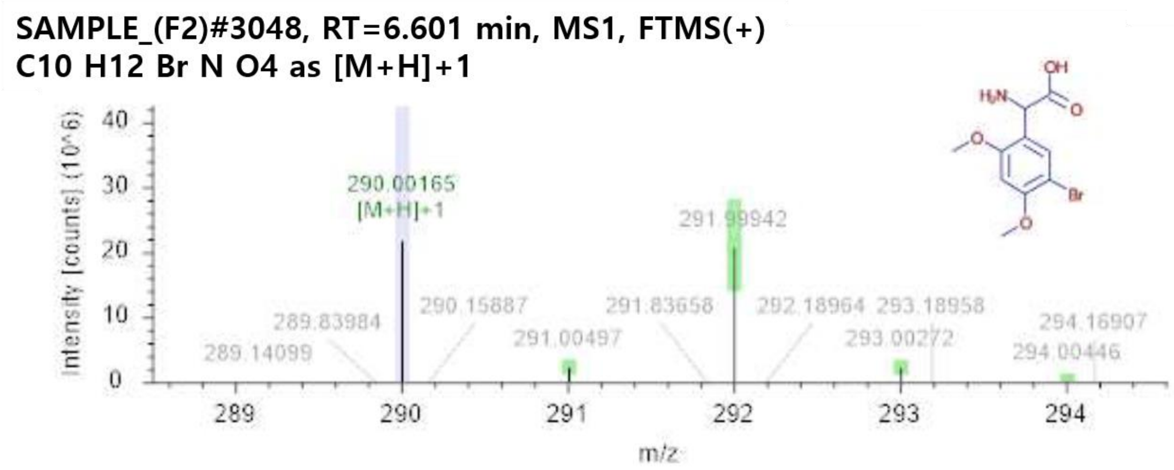

No. 8. D-(+)-Proline

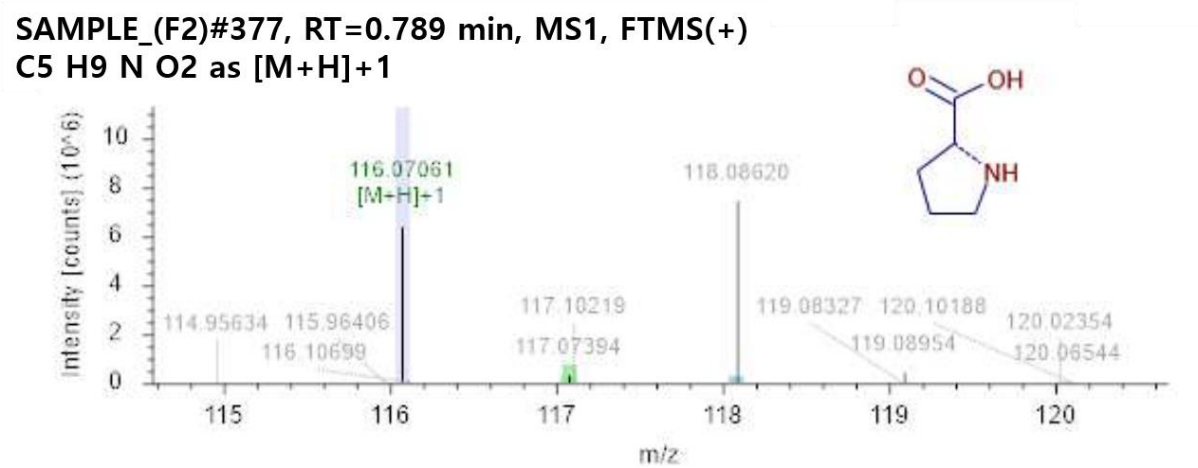

No. 9. 2-tert-Butyl-4-methoxyphenol

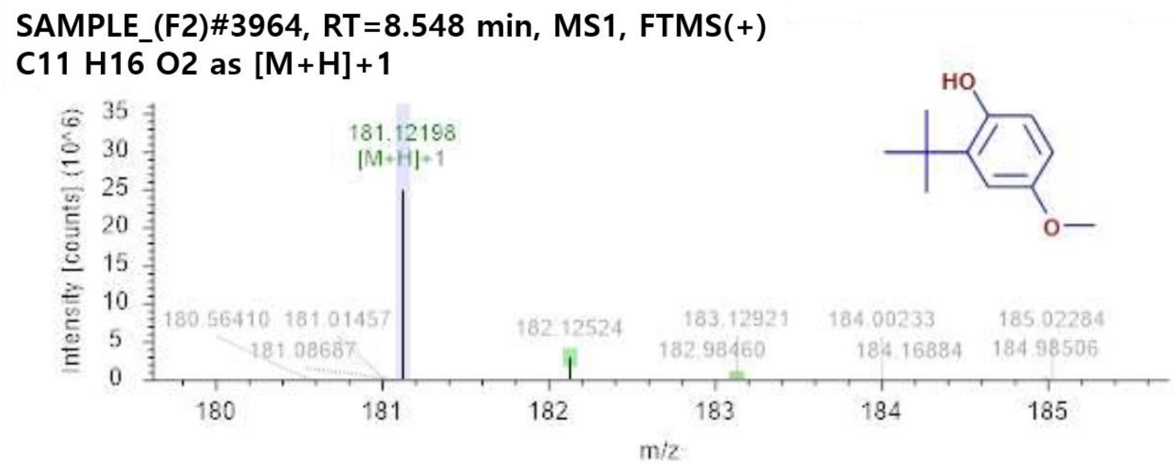

No. 10. Nicotinamide

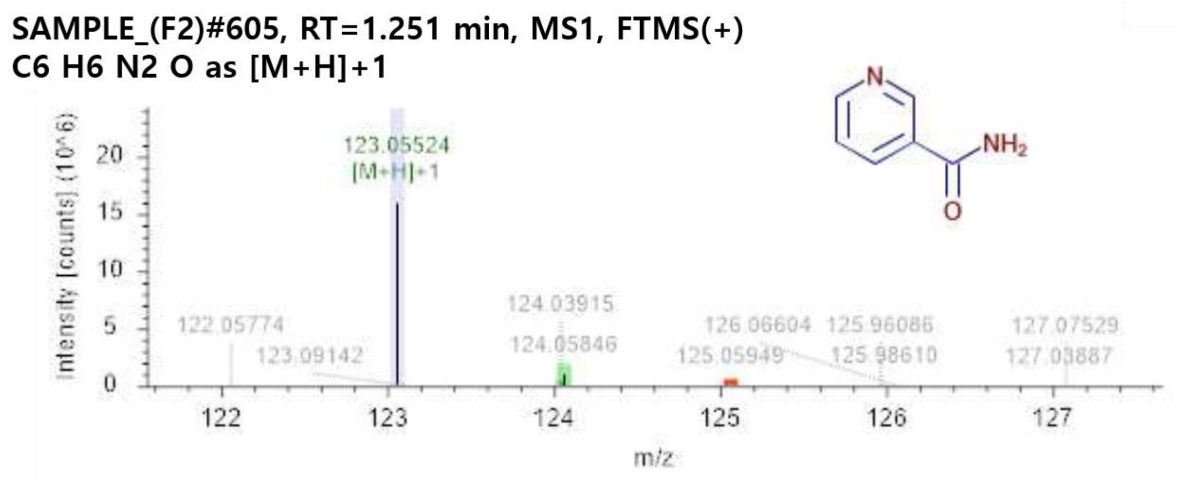

Table S1. LC/MS profile of 4 compounds with ≥ 90% match to the mzCloud spectral library

Name	mzCloud Best Match Confidence (%)	mzCloud Best Match (%)	ChemSpider Results	Area (Max.)
Nicotinamide	70.2	99.6	116	62495868.421
Erucamide	98.3	95.8	32	59229768.358
4-Guanidinobutyric acid	98.4	96	52	18905808.584
Monoolein	95.3	93.7	87	3714558.609

No.	Formula	Candidate M.W.	RT[min]
1	$C_6 H_6 N_2 O$	122.048	1.25
2	$C_{22} H_{43} N O$	337.3337	12.599
3	$C_5 H_{11} N_3 O_2$	145.0848	1.18
4	$C_{21} H_{40} O_4$	356.2922	10.778

Figure S3. Four candidate compounds were further screened on their anti-osteoclastic effect *in vitro.* (Left) TRAP staining shown in dark purple was observed at 40x magnification under a light microscope (Scale bar. 500 μm). (Right) Counts of multinuclear cells (≥3 nuclei) of TRAP-positive cells and larger multinuclear cells (≥10 nuclei) of TRAP-positive cells are presented as percentages. Data are shown as mean ± SEM (n≥3). One-way analysis of variance (ANOVA) was utilized, followed by Bonferroni post hoc test for pairwise comparisons. *$p < 0.05$; **$p < 0.01$; ***$p < 0.001$; n.s. = not significant.

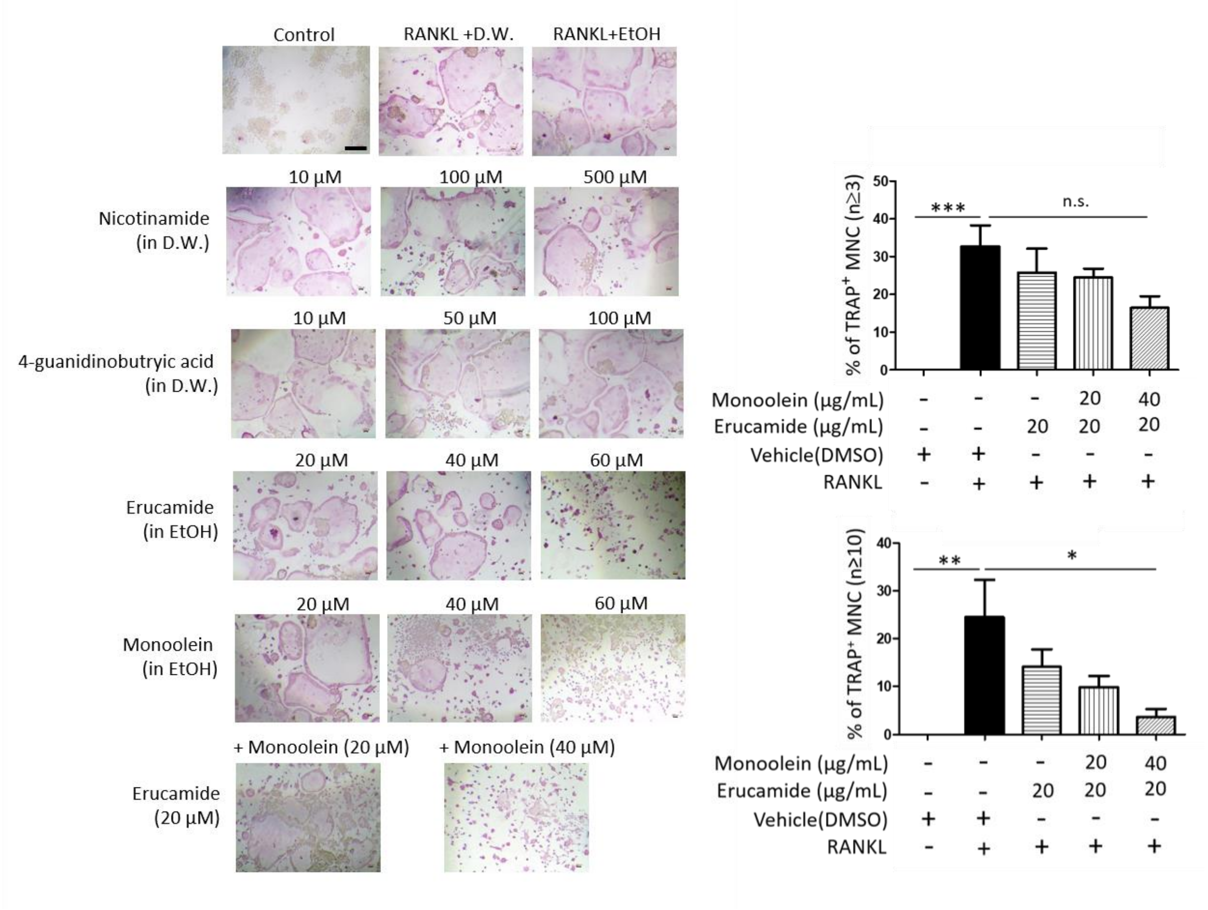

Supplement: Supplementary file 1 [file molecules-29-01741-s001.zip › molecules-2888145-supplementary.pdf]
